# White coat hypertension in pediatrics

**DOI:** 10.1186/s13052-016-0213-3

**Published:** 2016-01-15

**Authors:** Alexander Jurko, Milan Minarik, Tomas Jurko, Ingrid Tonhajzerova

**Affiliations:** Pediatric Cardiology Clinic, Jessenius Faculty of Medicine in Martin, Comenius University in Bratislava, Kollarova 2, 03601 Martin, Slovak Republic; Faculty of Health Care, Catholic University in Ruzomberok, Ruzomberok, Slovak Republic; Department of Neonatology, Jessenius Faculty of Medicine in Martin, Comenius University in Bratislava, Martin, Slovak Republic; Department of Physiology and Martin Centre for Biomedicine (BioMed), Jessenius Faculty of Medicine in Martin, Comenius University in Bratislava, Martin, Slovak Republic

**Keywords:** Hypertension, White coat, Blood pressure, Children, Cardiovascular

## Abstract

The article summarizes current information on blood pressure changes in children during clinic visit. White coat as a general dressing of physicians and health care personnel has been widely accepted at the end of the 19th century. Two problems can be associated with the use of white coat: white coat phenomenon and white coat hypertension. Children often attribute pain and other unpleasant experience to the white coat and refuse afterwards cooperation with examinations. Definition of white coat hypertension in the literature is not uniform. It has been defined as elevated blood pressure in the hospital or clinic with normal blood pressure at home measured during the day by ambulatory blood pressure monitoring system. White coat effect is defined as temporary increase in blood pressure before and during visit in the clinic, regardless what the average daily ambulatory blood pressure values are. Clinical importance of white coat hypertension is mainly because of higher risk for cardiovascular accidents that are dependent on end organ damage (heart, vessels, kidney). Current data do not allow any clear recommendations for the treatment. Pharmacological therapy is usually started in the presence of hypertrophic left ventricle, changes in intimal/medial wall thickness of carotic arteries, microalbuminuria and other cardiovascular risk factors. Nonpharmacological therapy is less controversial and certainly more appropriate. Patients have to change their life style, need to eliminate as much cardiovascular risk factors as possible and sustain a regular blood pressure monitoring.

## Background

White coat as a general dressing of physicians and health care personnel has been widely accepted at the end of the 19th century. It should represent a protection and feeling of cleanliness for the doctors and patients. Children often attribute pain and other unpleasant experience to the white coat and refuse afterwards cooperation with examinations. Significant number of pediatricians and pediatric nurses are therefore replacing white coat with casual dress in an attempt to improve patient’s cooperation. Still the majority of health care personnel are retaining white coat as traditional clothing. Two problems can be associated with the use of white coat: white coat phenomenon and white coat hypertension.

## Review

### Definition

The exact definition of white coat hypertension has been source of debates for a long time. White coat hypertension was described for the first time by Riva-Rocci more than 100 years ago. Initially, it has been considered as an abnormally elevated blood pressure in hospital or clinic with personnel wearing white coats. Later on, it has been shown that elevation of blood pressure can also be seen in an environment with health care personnel dressed casually [1]. Definition of white coat hypertension in the literature is not uniform. It has been defined as elevated blood pressure in the hospital or clinic with normal blood pressure at home measured during the day by ambulatory blood pressure monitoring system [2]. It is attributed to stress response to the physician or nurse and is often associated with increased heart rate. According to some authors white coat hypertension is present when mean blood pressure value is within the interval of hypertension and ambulatory blood pressure monitoring measured values are within normal limits [3]. White coat hypertension identifies patients who respond to stress with elevation of blood pressure. It is very likely, that these patients also respond with elevation of blood pressure to everyday stress [4].

## Specific aspects in pediatrics

Discussion about white coat hypertension in children has been going on since 1990 [5]. Similarly to adults, it is defined as elevated blood pressure in clinic with normal levels at home measured during the day with ambulatory blood pressure monitoring [1, 6].

White coat hypertension in children has also been considered as hypertension documented in clinic and concomitantly when both levels of systolic and diastolic pressures on daily ambulatory blood pressure monitoring were lower than the 95th percentile for height and sex or less than 135/85 mmHg for children older than 15 years [7].

Diagnosis of white coat hypertension can be made only after defining normal levels of blood pressure in the clinic. Arterial hypertension in children is defined as blood pressure equal or higher than the 95th percentile for age, sex and height recorded at 3 different measurements several weeks or months apart [8].

Because of the variability of high blood pressure in children it may be difficult to distinguish between physiologic and pathologic values. In order to avoid incorrect interpretation of blood pressure it is necessary to use percentile method and assess measured values according to age, height and sex [9]. Blood pressure measurement in clinic is the first step in evaluation of hypertension. Many authors, however suggest that ambulatory blood pressure monitoring is more accurate and is highly valuable method in evaluation of white coat hypertension, white coat effect and masked hypertension in children [4, 6, 10]. Despite the fact that ambulatory blood pressure monitoring can be associated with several negative emotional states of patients, such as discomfort accompanying the blood pressure measurement in periodical intervals leading to potential abnormal blood pressure values, ambulatory measurement of blood pressure is superior to clinic measurement in predicting cardiovascular mortality and nighttime blood pressure is the most potent predictor of outcome [11].

The most common mistake in ambulatory blood pressure results interpretation is the use of percentile graphs specified for single measurement or even values for adults [12].

With an increase in children obesity there is also increase in hypertension in children and adolescents. While prevalence of white coat hypertension in adults is between 11 and 57 % in dependence of criteria used, data on its prevalence in children are much more variable in literature. Prevalence in children with hypertension has been reported 47 % [7]. Comparable data are in another study with 45 % prevalence [13]. The highest reported prevalence of white coat hypertension in children was 60 % [14]. On the other hand, several articles describe much lower prevalence, in the range of 12,9 to 23,9 %. We have found 36,4 to 45 % prevalence in Slovakia [6, 10]. Different prevalence of white coat hypertension can be explained by regional demographic and anthropometric variabilities.

## Diagnostics

Ambulatory blood pressure monitoring confirmed that patient’s blood pressure in clinic may not represent his/her true blood pressure [15]. Ambulatory blood pressure monitoring is noninvasive, 24-hours continuous measurement of blood pressure using oscilloscopic method [16]. Several authors specify the methodology of ambulatory blood pressure monitoring [17–19]. Before initiation, casual blood pressure is obtained using appropriate cuff size after 5 minutes of quite sitting. We recommend to perform three consecutive measurements and the average value than represents the true blood pressure. Monitoring is done during regular working day and standard daily activities are allowed. Patient is connected to the monitoring system for 24 hours using pressure cuff usually on the nondominant extremity. Blood pressure is taken in 15 to 30 minutes intervals during day and in 30 to 40 minutes intervals at night (intervals can vary). Night time intervals are longer in order to decrease disruptions of patient’s sleep. Selection of appropriate cuff size is very important. It is done based on arm circumference measured in the half of the distance between ulnar olecranon a acromion. The arm circumference corresponds to the cuff length and corresponding width. Cuff width should be around 40 % of arm circumference. Minimal cuff width can also be calculated by multiplying the arm circumference (in cm) by 0.382. In case of borderline results, it is better to use wider cuff size because it does not significantly changes measured values [12]. The cuff should not cover cubital or axillary areas. It is desirable to have available 3 to 4 cuff sizes before monitoring. Inappropriately chosen cuff size can significantly modify obtained blood pressure values [20].

Important considerations during ambulatory blood pressure monitoring are [12]:

- use only internationally approved equipment
- use appropriate cuff size
- set up automatic measurement to maximum of 30 minute intervals to achieve adequate number of measurements
- cuff pressure release should not be faster than 2 mmHg/sec
- patient should maintain daily routine activities, avoid strenuous physical exercise and should remain quite during measurements
- patient or parents should take written notes of unusual events and provide information on the quality of sleep

The results should be evaluated by personnel with sufficient experience in pediatric hypertension [15].

## White coat effect

White coat effect and white coat hypertension are different in their definition, physiological mechanisms and clinical significance. White coat effect similarly to white coat hypertension depends on the stress response associated with visit in doctor’s office. In general it is blood pressure response to health care environment. It is defined as temporary increase in blood pressure before and during visit in the clinic, regardless what the average daily ambulatory blood pressure values are [17]. Some authors define white coat effect as a difference of 5 or more mmHg between the average blood pressure in the clinic and home environment [2, 7]. Basically, it is simple elevation of blood pressure during doctor’s visit that disappears afterwards regardless whether the patient is otherwise hypertensive or normotensive [15]. White coat effect and hypertension should not be used interchangeably. White coat effect is present in normotensive as well as hypertensive persons regardless whether they are on antihypertensive therapy or not [10, 21].

There is a theory that it is not only a direct response to blood pressure measurements [21]. This is supported by the evidence that elevated blood pressure:

- has been already observed before actual BP measurement
- is more pronounced when taken by physician than by nurse
- is not observed during automatic measurement

Exact pathophysiological mechanisms linking blood pressure response to stress factors are still not complexly explained.

White coat effect prevalence varies in different countries. Some authors consider it as a universal response occurring in majority, if not all, normotensive or hypertensive patients [22]. It is more frequent in women, elderly and patients with severe hypertension and obesity [2]. In children the frequency is quite high. It was found less frequent in normotensive patients in the age period from 6 to 15 years than in the age from 16 to 22 years. In hypertensive children, there was no significant difference within the age groups. This may suggest that white coat effect is more associated with hypertension than with age or physical activity [7].

## Risk factors

Several authors have tried to identify clinical, psychological a demographic predictors for white coat hypertension [17]. The results have been frequently inconclusive or statistically not significant. Following possible risk factors were suggested in some studies.

## Gender

Female gender is considered significant independent predictive factor for white coat hypertension [17, 23]. Similar results have been reported from pediatric studies [6, 7]. Because of small number of patients in pediatric studies, this information needs to be further elucidated.

## Familiar occurrence

Positive family history is a significant risk factor for white coat hypertension [6]. It is suggested that there is an association with 825 T alele of gene GNB 3 [24].

## Age

White coat hypertension in adults is associated with higher age. Some studies report five times higher incidence in elderly people [2]. Adult patient with diagnosed white coat hypertension were divided into 4 groups [23] (Table 1). In pediatric studies dependence on age has not been found [7, 14].

**Table 1:** White coat hypertension prevalence according to age

Age	White coat hypertension prevalence
<35 years	12 %
35 – 50 years	14 %
50 – 65 years	16 %
>65 years	17 %

## Body mass index

Obesity or high body mass index is considered a single correctable factor associated with primary hypertension [25]. Obesity in children and adolescents is becoming significant health problem. Incidence of obesity has increased three times over last two decades. One half of adults and every fifth child in Europe are overweight. One third from them are already obese and the percentage of obese children is increasing rapidly [26]. Pediatric studies confirmed statistically significant higher occurrence of white coat hypertension in obese children [14].

## Hypertension

Some authors consider prehypertension detected in clinic as a predictor of white coat hypertension [2, 27]. Higher incidence has also been found in adults with mild form of hypertension [17, 23]. In one pediatric study it has been shown that white coat hypertension is more frequently present in patients with essential hypertension [7]. Further studies are needed for confirmation that mild hypertension is a significant risk factor.

## Other risk factors

The frequency of clinic visits has been associated with prevalence of white coat hypertension in some studies. Less frequent clinic visits increase its prevalence [28]. In accordance with these results another study reported decrease the prevalence from 25.8 to 13.6 % from the first to fifth clinic visit. White coat hypertension also rises when blood pressure is taken by physician instead of nurse. It is therefore recommended that blood pressure in risk patient should not be taken by physician [2].

Other risk factors under consideration are smoking, prenatal malnutrition and low birth weight [29].

## Clinical importance

In the past, it has been considered benign condition. It may not have as significant sequelae as systemic hypertension but it should not be viewed as an unimportant issue [30]. Clinical importance of white coat hypertension is mainly because of higher risk for cardiovascular accidents that are dependent on end organ damage (heart, vessels, kidney) [31].

It has been shown in echocardiographic studies that left ventricular hypertrophy depends more on degree of blood pressure increase than on prevalence of high blood pressure [32]. When systolic ambulatory blood pressure is less than 120 mmHg the prevalence of left ventricular hypertension is very rare, with ambulatory blood pressure less than 130 mmHg it is around 6 % and at 140 mmHg it achieves 10 %. Patients with high blood pressure have higher prevalence of left ventricular hypertrophy than normotensive people, but lower than in hypertonics [2, 30, 33].

Important marker of cardiovascular risks associated with end organ damage is intimal/medial wall thickness of carotic arteries that can be measured by ultrasound. In one study it has been found that in normotensive people intimal/medial wall thickness was 0.76 mm, in patients with white coat hypertension 0.84 mm and in hypertensive patients 0.98 mm [2]. These results confirm that white coat hypertension is not a benign condition. Microalbuminuria has been observed in patients with high blood pressure which points to an association with renal tubular damage.

Cardiovascular risk factors are hypertriglycerinemia, hyperinsuilnemia and low HDL cholesterol levels.

Some authors speculate that white coat hypertension may be the initial stage in the development of systemic hypertension but this would require long term follow-up studies [6, 7, 34].

Current information suggests that it has better long term prognosis than systemic hypertension but is not completely harmless. In children it could represent an initial step to permanent systemic hypertension, so called prehypertensive stage [35]. Incidence of cardiovascular accidents for 100 patient year has been found to be 0.47 in normotensive people, 0.49 in patients with high blood pressure and 1.79 in patients with systemic hypertension. These authors also describe higher incidence of stroke in patients with white coat hypertension [2].

## Treatment

Approach to pharmacological treatment varies in different studies. Majority of authors do not recommend regular therapy [4]. On the other hand, there are authors who do not consider white coat hypertension as a benign condition and strongly recommend treatment [16]. Current data do not allow any clear recommendations for the treatment. Pharmacological therapy is usually started in the presence of hypertrophic left ventricle, changes in intimal/medial wall thickness, microalbuminuria and other cardiovascular risk factors [21].

Nonpharmacological therapy is less controversial and certainly more appropriate. Patients have to change the life style and eliminate as much cardiovascular risk factors as possible with regular blood pressure monitoring. They are recommended dietary salt restriction and weight reduction in case of overweight or obesity. Very important is regular physical activity, stopping smoking and restriction in use of alcoholic beverages. Appropriate education of children and their parents about medical complications of obesity is necessary. Diet should contain low fat and cholesterol products and include cereals, fruits and vegetables. Short term diets and exercises with rapid weight loss should be avoided. Appropriate measures are regular physical activity lasting 30 to 60 minutes 3 times weekly. Very helpful may be cooperation with pediatric psychologist. Blood pressure has to be regularly monitored every 6 to 12 months in order to detect development of permanent hypertension [7, 10].

## Conclusions

Clinical importance of white coat hypertension is mainly because of higher risk for cardiovascular accidents that are dependent on end organ damage (heart, vessels, kidney). Current data do not allow any clear recommendations for the treatment. Pharmacological therapy is usually started in the presence of hypertrophic left ventricle, changes in intimal/medial wall thickness of carotic arteries, microalbuminuria and other cardiovascular risk factors. Nonpharmacological therapy is less controversial and certainly more appropriate. Patients have to change the life style and eliminate as much cardiovascular risk factors as possible with regular blood pressure monitoring.
